# Supplementary material for: Study on the measurement of industrial eco-efficiency, spatial distribution and influencing factors in Yangtze River Economic Belt
Source: PLoS One. 2023 Apr 7;18(4):e0283964. doi: 10.1371/journal.pone.0283964 (PMC10081804; doi:10.1371/journal.pone.0283964)
Supplement: Attachment — Submitted filename: Response to Reviewer 4 Comments.doc [file pone.0283964.s002.doc]

**Response to Reviewer 4 Comments**

Dear editor and reviewers:

Thank you for taking time out of your busy schedule to review the manuscript. Now we have carefully corrected and replied the manuscript for this revision. The revision instructions are as follows:

**1.Comment:** In the abstract, the authors explain the background of the study and the main contents and conclusions of the study, but a sentence needs to be added to the abstract to clearly explain the necessity of this study.

**Response:** Thank you for your comment. We have realized that this necessity may not have been expressed clearly enough in the abstract section of the previous manuscripts. In lines 20-23, we have rewritten the research necessity and significance of the abstract.

**2.Comment:** In the introduction the authors describe the background and significance of the study, but a sentence or two needs to be added to clearly explain the innovation and specific contribution of the study.

**Response:** Thank you for your comment. We have presented the marginal contribution of this paper in the last paragraph of the literature review section, please refer to lines 149-159.

**3.Comment:** It is reasonable to select input indicators from three aspects: economy, environment and resources, but some references need to be added in this paragraph (line231-line239).

**Response:** Thank you for your comment. We have realized this issue and have revised the manuscript, please refer to lines 245-248.

**4.Comment:** The text annotations below the table are used to supplement the content of the table, but the annotations about the table please indicate for example lines 267 and 490.

**Response:** Thank you for your comment. We have carefully examined the tables in the full text and the only ones that involve the need to add sub-table text comments are Tables 2 and 6.

**5.Comment:** The data in the table are calculated from the model and the contents of the table should be explained to facilitate understanding. For example, the meaning of the values represented by each column in Table 4.

**Response:** Thank you for your comment. In lines 379-385 of the manuscript, we have explained the meaning of the data derived in Table 4

**6.Comment:** The punctuation in the article is consistent with the need to make reasonable use of it so as not to affect the reading. Please check the punctuation throughout the article for correctness, such as the semicolon in line 339.

**Response:** Thank you for your comment. We have corrected the place you raised and double-checked the whole text to correct the unreasonable symbols.

**7.Comment:** The article uses a table to show the names and units of the indicators, which is very clear and concise. However, it is important to note that the case of the words in each phrase in the table is consistent. Please check the spelling of the words in each table to make sure that the case in each table is consistent. e.g. 'billion cubic meters' in Tab.1.

**Response:** Thank you for your comment. We have made uniform changes to the words in the form.
